# Treatment of Asphyxia from Drowning and Hanging

**Published:** 1851-11

**Authors:** 


*Treatment of Asphyxia from Drowning and Hanging.* From DR. R. H. STORER'S Address before the Massachusetts Medical Society.

How little understood, among many of the well-educated and intelligent in our community, is the treatment for the restoration of the drowned! How many lives must have been sacrificed by the barbarous custom of suspending the asphyxied by the feet, or rudely rolling them upon barrels with the head dependent, for the purpose of freeing the lungs of the water with which they were supposed to be filled — a custom which, within a few years, has fallen under my immediate observation.

How many, apparently dead, have been restored to their afflicted friends by means of long-continued, scientific efforts; by having their bodies carefully dried, and exposed to a moderate temperature — their heads and shoulders elevated — their lungs artificially inflated; by the exhibition of external and internal stimulants, and judicious venesection!

How many have thus been resuscitated, after all human means seemed unavailing — long after the bystanders have ceased their efforts, and none, save the almost frantic parent or child, have in silent prayer continued their exertions! Numerous cases might be cited to show that life has been recalled after a body has been immersed for a very long period. These instances should cheer the desponding, and encourage them to labor while there seems the slightest possibility of restoration. Allow me to illustrate this remark with a single example, which was published during the last year in the "Northern Lancet and Gazette of Legal Medicine." It was communicated by Charles McNeil, Esq., of Charlotte, Vt., and is the touching story of a grateful father. "One of my sons, nine or ten years of age, was on Sunday afternoon, in August, 1830, found to be missing. On inquiry, I ascertained that he had last been seen playing on a boat lying at the wharf. The day was calm, and the waters of Lake Champlain still and unruffled by a ripple; but, knowing that he had been on the boat, his brother was sent to search for him, but he returned without any tidings. Once more he returned to the boat, and, looking carefully in every direction, discovered him lying on the bottom of the lake in eight feet water, where he must have lain half an hour, if not longer, when he was brought to the surface. I received the body: it was rigid and cold, as also were the limbs; a bluish cast was spread over the countenance; the deep solicitude of a father discovered no signs of life — no heat; the heart was stilled and the lungs quiescent. No more would I have anticipated the presence of life, if he had been submerged for several years; and had I not, some days previous to the accident, providentially read in an old paper an article by Dr. Buchanan, of Philadelphia, on the subject of restoring suspended animation after submersion, we should have consigned the body to the grave, as it was recovered from the lake. The body being placed on a bed, some of the neighbors were directed to rub it briskly with flannel cloths — an order which they obeyed with great reluctance, from the thought of performing this office on a corpse; and I will admit that I somewhat entertained the same opinion. Still, I would fain hope, and urged on my friends the continuance of their exertions; the friction was persevered in; warm flannel sheets were applied in rapid succession. This treatment was continued for thirty or forty minutes, when we were gratified by hearing a feeble murmur in the throat, followed soon after by a slight quivering of the lips. The case, however, was enveloped in doubt and obscurity for a long time, as the recovery was extremely slow."

The above remarks might, with equal propriety, be applied to the subject of hanging. Many judicious general practitioners entertain the most vague and unsatisfactory notions regarding its phenomena. They not only are unacquainted with the several appearances produced in individual cases of suspension, but they really are not aware how death is produced; and, cerebral apoplexy not unfrequently being considered the cause, copious depletion, employed instead of artificial respiration, checks the vital current forever.— *Boston Med. and Surg. Jour.*

